# Genome-Wide Identification of DNA Methylases and Demethylases in Kiwifruit (*Actinidia chinensis*)

**DOI:** 10.3389/fpls.2020.514993

**Published:** 2020-09-09

**Authors:** Yaoxin Zhang, Xiaoqing He, Haochen Zhao, Wencai Xu, Heng Deng, Huan Wang, Shuyue Wang, Dan Su, Zhenlei Zheng, Bin Yang, Don Grierson, Jun Wu, Mingchun Liu

**Affiliations:** ^1^Key Laboratory of Bio-Resource and Eco-Environment of Ministry of Education, College of Life Sciences, Sichuan University, Chengdu, China; ^2^Sichuan Dexin Guoyuan Biological Technology Co., Ltd., Wenchuan, China; ^3^School of Biosciences, University of Nottingham, Sutton Bonington Campus, Loughbotrough, United Kingdom

**Keywords:** DNA methylases, C5-MTases, DNA demethylases, *Actinidia chinensis*, kiwifruit

## Abstract

DNA methylation plays an important role in a wide range of developmental and physiological processes in plants. It is primarily catalyzed and regulated by cytosine-5 DNA methyltransferases (C5-MTases) and a group of DNA glycosylases that act as demethylases. To date, no genome-scale analysis of the two kiwifruit (*Actinidia chinensis*) families has been undertaken. In our study, nine C5-MTases and seven DNA demethylase genes were identified in the kiwifruit genome. Through selective evolution analysis, we found that there were gene duplications in C5-MTases and demethylases, which may have arisen during three genome doubling events followed by selection during evolution of kiwifruit. Expression analysis of DNA methylases (C5-MTases) and demethylases identified changes in transcripts of DNA methylation and demethylation genes during both vegetative and reproductive development. Moreover, we found that some members of the two methylase/demethylase families may also be involved in fruit ripening and the regulation of softening. Our results help to better understand the complex roles of methylation/demethylation in plants and provide a foundation for analyzing the role of DNA methylation modification in kiwifruit growth, development and ripening.

## Introduction

The accurate qualitative, quantitative and temporal regulation of gene expression accomplished by *cis*-regulatory elements and *trans*-acting factors is indispensable for normal development in plants and animals. In addition to the conventional genetic basis of inheritance and gene expression, epigenetic processes cause heritable changes in gene function that occur by covalent modification of DNA without alteration in the base sequence (Holliday, 2006). One major epigenetic modification is DNA methylation, which occurs by the addition of methyl groups to the C-5 site of cytosine, the N-6 site of adenine, and the N-7 site of guanine (Jeltsch, 2002) in DNA molecules. These epigenetic regulatory mechanisms are widely found in plants and animals (Cao et al., 2014; Wang et al., 2016) and play an important role in the regulation of gene expression. Previous studies have shown that DNA methylation of cytosine at the c-5 site is important in various biological processes, including genome stability, gene imprinting, growth and development, stress response, and biosynthetic regulation of secondary metabolites (Hsieh and Fischer, 2005; FitzGerald et al., 2008; Yelina et al., 2015). In plants, DNA methylation occurs primarily in three distinct sequence contexts: symmetric CG, CHG, and asymmetric CHH sites (where H =A, T or C) (Law and Jacobsen, 2010).

Dynamic mechanisms of DNA methylation in plants include: *de novo* methylation, DNA methylation maintenance, and DNA demethylation. Cytosines in all sequence contexts can be *de novo* methylated through the well-known RNA-directed DNA methylation pathway (RdDM), in which 24-nt siRNAs guide the DNA methyltransferase domains rearranged methyltransferase 2 (DRM2) to methylate target loci (Matzke and Mosher, 2014). In *Arabidopsis*, pathways have been identified that indicate DNA methyltransferase 1 (MET1), as well as chromomethylase 3 (CMT3), can maintain CG and CHG methylation states, whereas chromomethylase 2 (CMT2) can maintain CHH methylation (Law and Jacobsen, 2010). The dynamic regulation of DNA methylation levels also requires the participation of DNA demethylases, which can excise 5-meC from sequence contexts (Zhu, 2009). Four DNA demethylases have been reported in *Arabidopsis*, namely Repressor of Silencing 1 (ROS1), DEMETER (DME), DEMETER-LIKE (DML2) and DML3 (Gong et al., 2002; Gehring et al., 2006; Ortega-Galisteo et al., 2008).

Kiwifruit (*Actinidia chinensis*) is an important economic crop and a member of the *Actinidiaceae* family that consists of three genera and approximately 360 species (Huang et al., 2013). In other plants, DNA methylation has been shown to be involved in many aspects of plant growth and development, such as resistance response, leaf polarity, flowering time, fruit ripening and resistance to disease (Hsieh and Fischer, 2005; FitzGerald et al., 2008; Yelina et al., 2015). The importance of methylation has been demonstrated, for example, by the finding that DNA methylase inhibitors induce early ripening of tomato fruit (Zhong et al., 2013). In contrast, absence of demethylase gene function delays the ripening process (Lang et al., 2017). Despite the fact that C5-MTases and DNA demethylases play a fundamental role in the determination of DNA methylation pattern in the epigenome, little is known about the control of DNA methylation status in many plants, including kiwifruit. To investigate the putative role of DNA methylation in kiwifruit developmental process, we first identified C5-MTases and DNA demethylases family genes in the genome.

In our study, the protein sequences of C5-MTases and DNA demethylases across the whole genome of kiwifruit (Pilkington et al., 2018) were determined and the phylogenetic relationship among C5-MTases and demethylases in kiwifruit were analyzed. We also measured the expression of C5-MTase and demethylase encoding genes in various tissues/developmental stages in kiwifruit. In addition, to further understand the functions of selected members, three-dimensional (3D) structural modeling was performed. Our results provide a foundation for analyzing the role of DNA methylation modification in kiwifruit.

## Materials and Methods

### Data Collection and Identification of C5-MTases and DNA Demethylases in Kiwifruit

Protein sequences of C5-MTases and DNA demethylases in *Arabidopsis* (Gong et al., 2002; Xiao et al., 2006; Henderson et al., 2010; Liu et al., 2015) were downloaded from the National Center for Biotechnology Information (NCBI) database (http://www.ncbi.nlm.nih.gov/). Gene IDs for all protein sequences are shown in **Supplementary Data 1**. Using Blastp (2.9.0) (E < 0.001) (Camacho et al., 2009), putative protein sequences from the whole genome of kiwifruit (Pilkington et al., 2018) with homology to methylase and demethylase families in *Arabidopsis* were identified. We then used Hmmsearch (3.2.1) (Wheeler and Eddy, 2013) to scan all previously identified sequences (Gong et al., 2002; Xiao et al., 2006; Henderson et al., 2010; Liu et al., 2015). Finally, we identified nine C5-MTases and seven DNA demethylases. Furthermore, names to C5-MTases and DNA demethylases in kiwifruit were assigned based on names of best hit proteins by NCBI-blastp (Non-redundant protein sequences database) (Letunic et al., 2015). The isoelectric point (pI) and molecular weight (Mw) of all C5-MTases and DNA demethylases proteins were analyzed by the online program Expasy (http://web.expasy.org/compute_pi/) (Gasteiger et al., 2005).

### Analysis of Conserved Motif, Domain, Gene Structure, and 3D Model

Full length amino acid sequences of C5-MTases and DNA demethylases in kiwifruit were analyzed using the MEME (5.05) program (http://meme.nbcr.net/meme/) to study their conserved motifs (parameter settings: pattern 10; minimum pattern width 6; maximum pattern width 50). Both Pfam (32.0) (http://pfam.xfam.org/) and SMART (http://smart.embl-heidelberg.de/) were used for protein domain identification in kiwifruit, *Arabidopsis*, tomato, and rice. Tbtools was used to draw the gene structure of the C5-MTases and DNA demethylases from the kiwifruit genome (Chen et al., 2018). SWISS-MODEL (http://www.swissmodel.expasy.org/interactive) was used for building a AcCMT3, AcCMT4, AcCMT5, and AtCMT1 homologous protein model (at least 186 models for each protein were generated using the “building model” engine, and the best model was selected based on the best global model quality estimation) (Guex et al., 2009; Benkert et al., 2011; Bienert et al., 2016; Bertoni et al., 2017; Waterhouse et al., 2018).

### Multiple Sequence Alignment and Phylogenetic Tree Construction

Multiple sequence alignments of the C5-MTase and DNA demethylase amino acid sequences were performed with MEGA X (10.0.5) (Kumar et al., 2018). Neighbor-Joining (NJ) and maximum likelihood (ML) trees were constructed using MEGA X (10.0.5) with aligned protein sequences (Poisson correction and bootstrap = 1000 replicates) (Yang, 2007). Visualization of the phylogenetic tree was accomplished by JalView (Waterhouse et al., 2009; Troshin et al., 2011) and Evolview v2 (https://www.evolgenius.info/) (Zhang et al., 2012; He et al., 2016).

### Analysis of Selection Pressure

Blastp (E<1e-5) and MCScanX (Wang et al., 2012) were used for colinear analysis. Visualization of the data was accomplished by Circos plot (Connors et al., 2009). We used DnaSP (v6.12.03) (Rozas et al., 2017) to calculate the Ka/Ks values of the corresponding C5-MTases and DNA demethylases in kiwifruit.

### Analysis of the Promoter Cis-Regulating Elements

The 2000 bp of kiwifruit genomic DNA sequence upstream of the transcriptional start sites of C5-MTases and DNA demethylases were extracted from the kiwifruit genome. The online tool PlantCare (http://bioinformatics.psb.ugent.be/webtools/plantcare/html/) (Lescot et al., 2002) was used to analyze the promoter sequences.

### Analysis of Gene Expression

A plant RNA extraction kit (V1.5) (DNase I) (Chengdu Biofit Biotechnologies CO., LTD) was used to extract the total RNA of roots, young stems, stems, young leaves, leaves, flowers, fruits of 40 days after anthesis, fruits of 140 days after anthesis, fruits of 4 days after harvest, and fruits 12 days after harvest of kiwifruit. The roots, young stems and young leaves were obtained from tissue-cultured seedlings. cDNA was obtained by reverse transcription according to the PrimeScript™RT reagent Kit with gDNA Eraser (Perfect Real Time) (Takara biomedical technology (Beijing) co., LTD., Beijing, China). Real-time quantitative (RT) PCR was performed as described by Pirrello et al., 2006 (Pirrello et al., 2006). AcActin1 (EF063572) genes (Wang et al., 2019) were used as a standardized internal control and relative mRNA levels of genes were calculated as 2-ddct values. Primers for amplification were designed using the software PerlPrimer v1.1.21 (Marshall, 2004) (**Supplementary Data 2**). Three independent biological replicates were used for this experiment.

## Results

### Identification and Analysis of C5-MTase and DNA Demethylase Genes in Kiwifruit

In our study, C5-MTases and DNA demethylases were identified from the kiwifruit genome using the sequences of C5-MTases and DNA demethylases of *Arabidopsis* as BLAST queries against the kiwifruit genome. We then used HMMER to verify whether the identified C5- MTases contained a typical DNA methylase domain (PF00145). By using this method, nine C5-MTase and seven DNA demethylase genes were identified in kiwifruit (**Table 1**). The number of genes is similar to that in *Arabidopsis* and tomato species (Gong et al., 2002; Xiao et al., 2006; Hsieh et al., 2009; Henderson et al., 2010; Cao et al., 2014). The polypeptide lengths of identified C5-MTase genes (CMT1, CMT2, CMT3, CMT4, CMT5, DRM2X1, DRM2X2, DRM2X3, and MET) in kiwifruit ranges from 558 to 1055 amino acids and the predicted molecular weights are from 63.11 to 119.70 (kDa). The seven demethylase genes (DME1, DME2, DME3, DME4, DML, ROS1X1, and ROS1X2) code polypeptides composed of 1202 to 1980 amino acids. The molecular weights of these demethylases ranges from 135.51 to 220.26 (kDa).

**Table 1:** Basic Information of C5-MTase and DNA Demethylase Genes in kiwifruit.

Protein name	Gene ID	Chromosome_location			Protein			ORF length(bp)
					PI	Mw(kDa)	Length(aa)	
**Cytosine-5 DNA methyltransferases**								
MET	Acc06669	Chr6	11943186	11957240	5.69	101.17	902	2706
CMT1	Acc10156	Chr9	4688362	4699999	5.46	93.73	830	2490
CMT2	Acc22670	Chr20	4250119	4266395	6.44	119.70	1055	3165
CMT3	Acc29050	Chr25	16658886	16677120	6.45	105.67	923	2769
CMT4	Acc30443	Chr26	19829011	19840660	5.15	92.97	821	2463
CMT5	Acc32510	Chr28	15169385	15181698	5.06	105.30	928	2784
DRM2X1	Acc02430	Chr2	13147187	13161342	5.13	66.53	591	1581
DRM2X2	Acc05665	Chr5	11554590	11564191	5.87	63.11	558	1674
DRM2X3	Acc30617	Chr27	1537771	1548409	5.33	65.84	585	1554
**Demethylases**								
DME1	Acc04130	Chr3	18269904	18284006	8.23	220.26	1980	5940
DME2	Acc08438	Chr8	1986494	1997536	6.33	189.93	1706	5118
DME3	Acc27569	Chr24	9270345	9285217	8.3	216.44	1953	5859
DME4	Acc16912	Chr22	13533612	13546816	6,15	208.53	1868	5604
DML	Acc00310	Chr1	3905070	3916337	9.24	158.76	1405	4215
ROS1X1	Acc03168	Chr3	6425862	6443713	9.31	138.30	1223	3669
ROS1X2	Acc08944	Chr8	11005987	11014368	9.26	135.51	1202	3606

**Figure 1:**
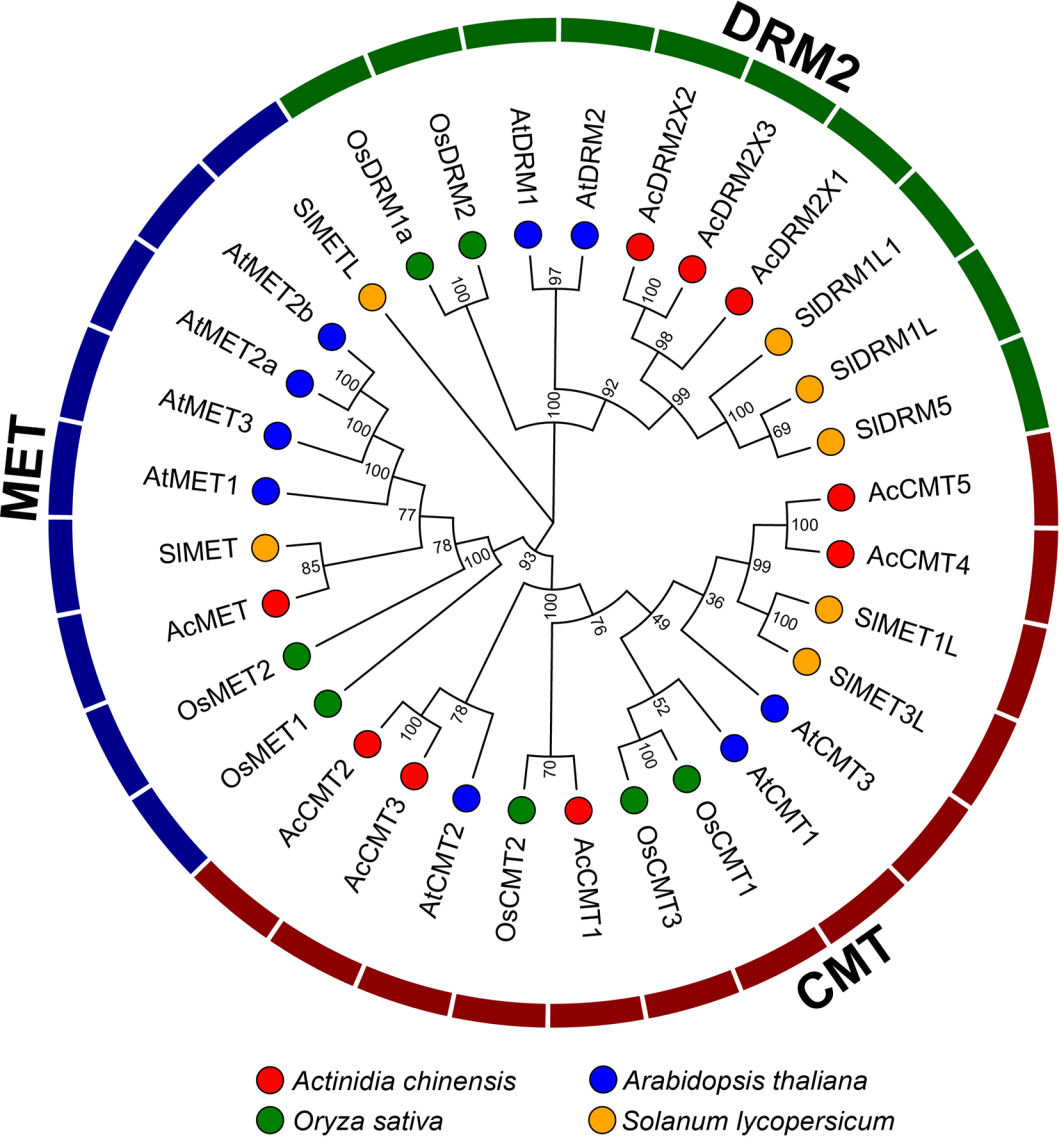
Neighbor-joining phylogeny of C5-MTases.

To investigate the phylogenetic relationship of C5-MTase and DNA demethylase genes in kiwifruit, we constructed a phylogenetic tree using the neighbor-joining (NJ) method based on multiple sequence alignments (**Figure 1**). The results showed that the C5-MTase families of the four plants are divided into three distinct branches, namely CMT, MET, and DRM2. The evolutionary branch of CMT contains five kiwifruit proteins (CMT1, CMT2, CMT3, CMT4, and CMT5), while the evolutionary branch of MET contains a single protein in kiwifruit (MET), and the evolutionary branch DRM contains three proteins in kiwifruit (DRM2X1, DRM2X2, and DRM2X3).

Interestingly, C5-MTases in kiwifruit were found to be closer to that in tomato. Moreover, it was also found that proteins in dicotyledonous plants (kiwifruit, *Arabidopsis*, and tomato) and monocotyledon (rice) are significantly different. These results suggested that the duplication of C5-MTases might be different between dicotyledonous and monocotyledonous plants.

**Figure 2:**
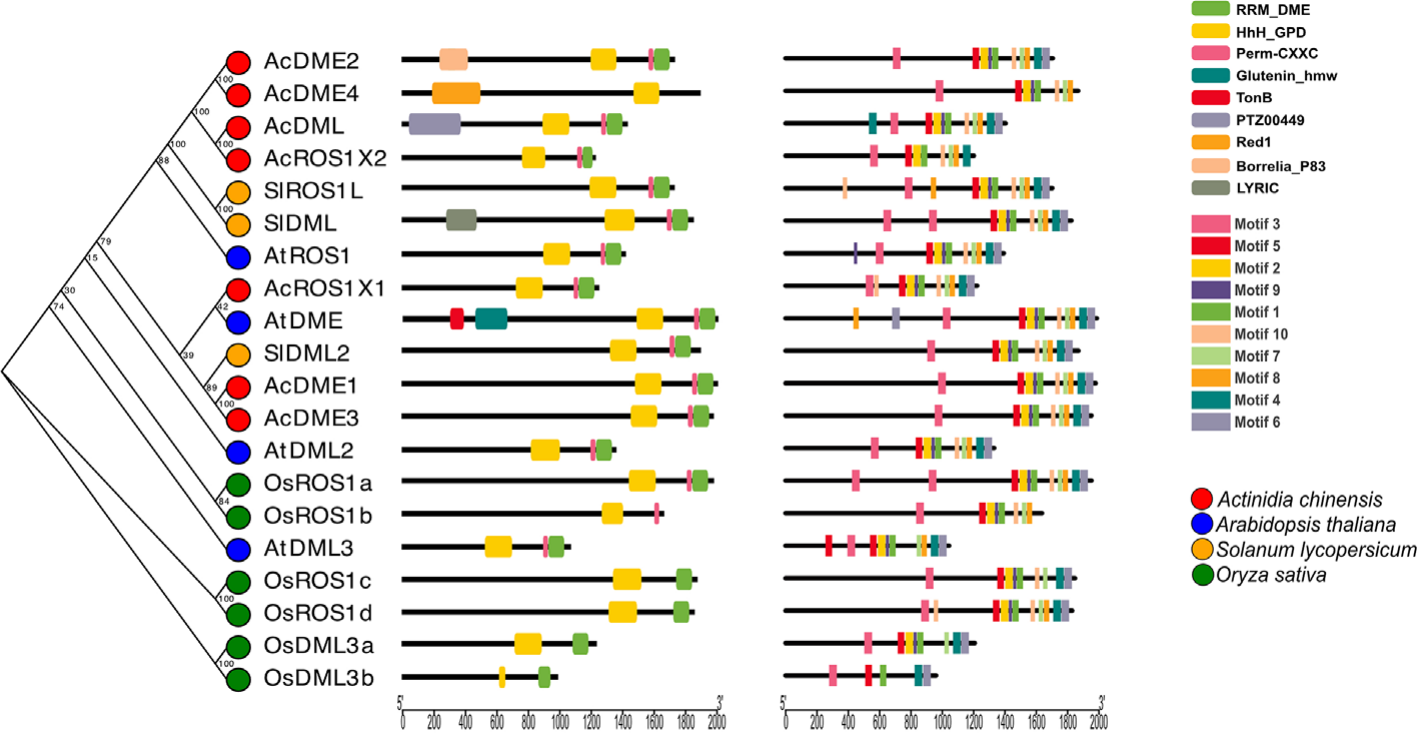
DNA demethylase gene structure, phylogenetic relationships and conserved motifs in four species.

The characteristic conserved key domain of demethylase proteins is HhH_GPD and most also possess RRM_DME and Perm-CXXC domains (**Figure 2**). For further analysis of the evolutionary relationships among plant demethylase families, we constructed a rootless evolutionary tree using 20 full-length protein sequences from four plant species (kiwifruit, *Arabidopsis*, tomato, and rice) and analyzed their motifs. We found that DNA demethylases share similar conserved domains (**Figure 2**), indicating that the demethylase gene family has been relatively conserved in different plant species. However, the monocotyledons and dicotyledons are clearly separated in the evolutionary tree, indicating that the evolution of DNA demethylase might have occurred separately in monocotyledons and dicotyledons. This is consistent with previously constructed phylogenetic trees using the DNA glycosylase domain of DMLs from flowering plants (Zemach et al., 2010). These results also showed the conservation and diversity of DNA demethylase between monocotyledons and dicotyledons.

### Motif and Domain Analysis of C5-MTases and DNA Demethylases in Kiwifruit

We found that members of the CMT protein group not only contain a DNA methylase domain, but also a chromosome domain located in the DNA methylase domain and a bromine-adjacent homologous domain (BAH). The MET group members include a DNA-methylase domain and two bromine-adjacent homologous domains (BAH) (Xiao et al., 2006; Henderson et al., 2010; Song et al., 2013; Cao et al., 2014; Wang et al., 2016). The seven demethylase genes all contain a HhH_GPD domain, a RRM_DME domain, and a Perm-CXXC domain, except DME4 which only has a HhH_GPD domain (**Supplementary Data 3**) (Gong et al., 2002; Liu et al., 2015; Wang et al., 2016).

**Figure 3:**
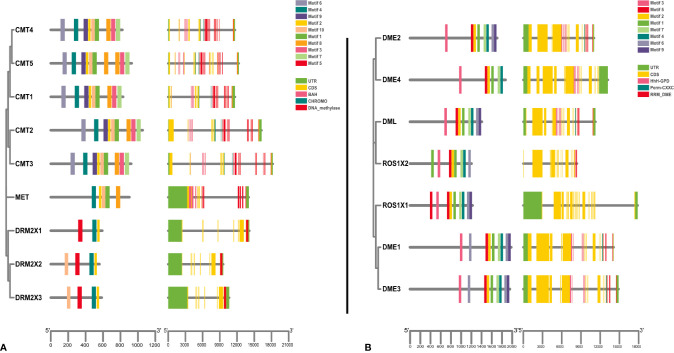
Gene structure of kiwifruit C5-MTase and demethylase genes and phylogenetic relationships between conserved motifs of each group of kiwifruit proteins. **(A)** C5-MTase; **(B)** Demethylase.

To further explore the conservation and diversity of C5-MTases and DNA demethylases in kiwifruit, we used MEME to analyze their protein motifs (E ≤ 0.01) (**Supplementary Data 4**). All C5-MTase genes were found to contain motif 2 and motif 4. Specifically, all members of the MET group contained motif 1, motif 2, motif 4, motif 8, and motif 10. All members of the CMT group contained motif 1, motif 2, motif 3, motif 4, motif 6, motif 7, Motif 8, motif 9, and motif 10. All members of the DRM group contained motif 2, motif 4, and motif 5 (**Figure 3**). As shown in **Figure 3A**, the conservation of C5-MTases amino acid sequences in kiwifruit was higher than that the DNA sequence. All DNA demethylase genes contain motif 1, motif 2, motif 3, motif 4, motif 5, and motif 8, which demonstrates a high degree of sequence conservation (**Figure 3B**).

### Homology Analysis of C5-MTases and DNA Demethylases in Kiwifruit

**Figure 4:**
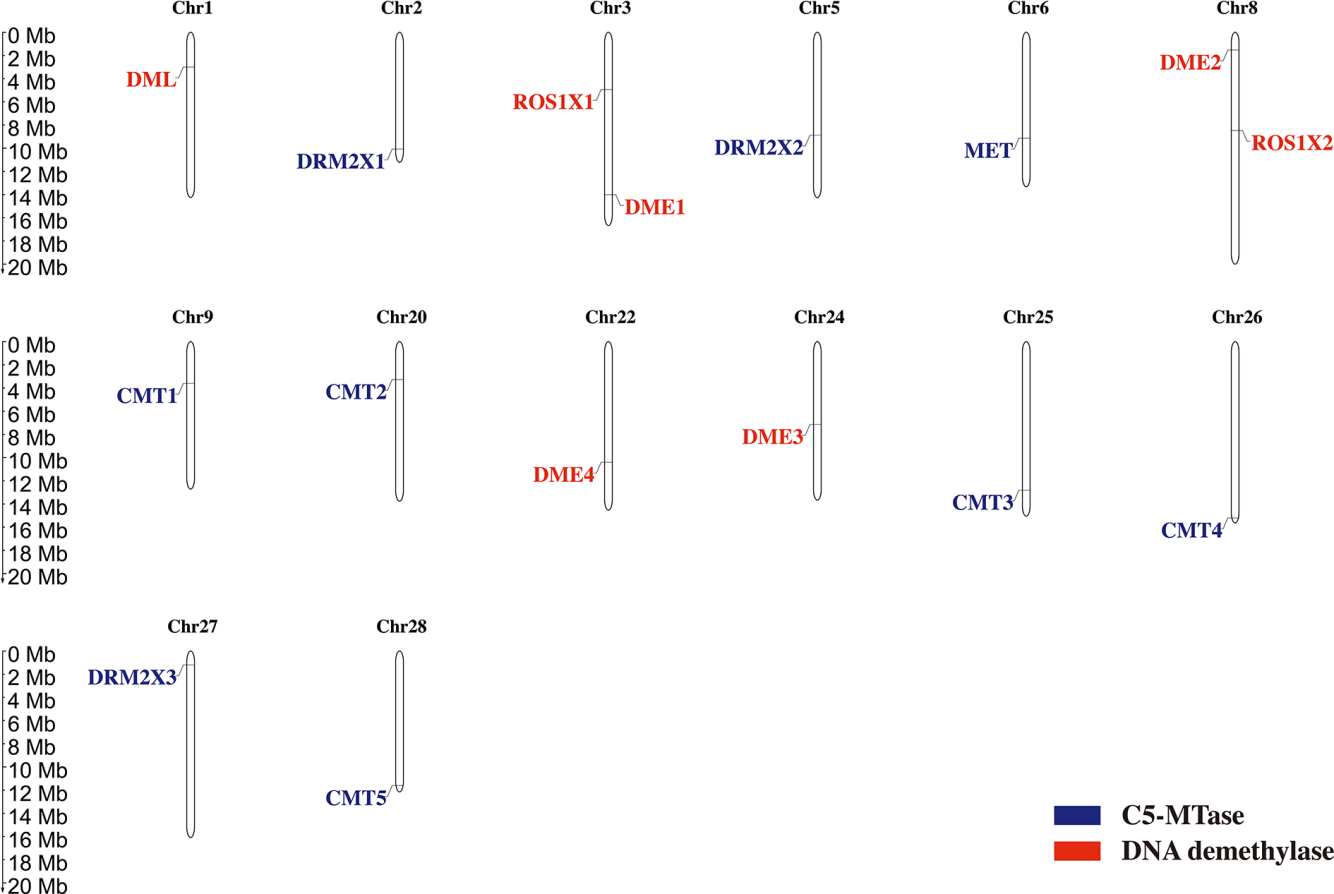
Chromosome localization of C5-MTase and demethylase genes in kiwifruit.

The distribution of genes across chromosomes can influence their downstream functions. The location of C5-MTases and DNA demethylases in kiwifruit chromosomes were determined according to the genome and gene annotation (**Figure 4**). Nine C5-MTase genes in kiwifruit are dispersed across chromosome 2 (DRM2X1), chromosome 5 (DRM2X2), chromosome 6 (MET), chromosome 9 (CMT1), chromosome 20 (CMT2), chromosome 25 (CMT3), chromosome 26 (CMT4), chromosome 27 (DRM2X3), and chromosome 28 (CMT5). Seven kiwifruit DNA demethylase genes are dispersed across chromosome 1 (DML), chromosome 3 (ROS1X1 and DME1), chromosome 8 (DME2 and ROS1X2), chromosome 22 (DME4), and chromosome 24 (DME3).

**Figure 5:**
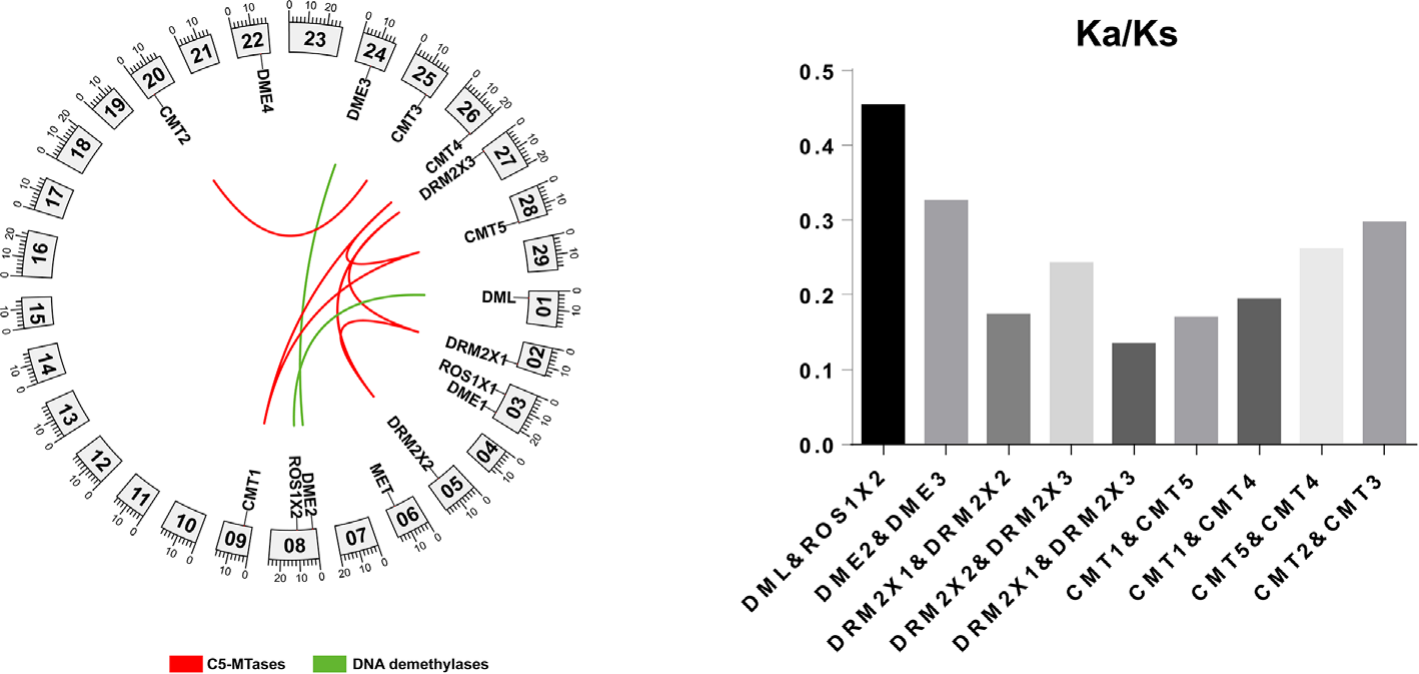
Homology analysis of C5-MTases and DNA demethylases.

The potential mechanisms and evolutionary relationships between C5-MTases and DNA demethylases in the kiwifruit genome were studied using Blastp. The results were analyzed by colinearity analysis using MCScanX as shown in **Figure 5**. Five groups of genes were found to possess colinearity including DRM2X1, DRM2X2, and DRM2X3; CMT1, CMT4, and CMT5; CMT2 and CMT3; DME2 and DME3; DML and ROS1X2. The level of colinearity in C5-MTases (33.3%) was close to that of DNA demethylases (28.6%). The emergence of these homologous gene pairs may be associated with three gene doubling events that have occurred in kiwifruit (Huang et al., 2013).

To analyze the constraints that have governed C5-MTases and DNA demethylases evolution in kiwifruit, we investigated the Ka/Ks ratios for five duplicated pairs of genes. The results showed that all Ka/Ks ratios are lower than 1.0 (**Figure 5**), which suggested that these homologous genes have undergone purifying selection (Zhang et al., 2006).

### *Cis*-Acting Elements Analysis of C5-MTases and DNA Demethylases Promoters in Kiwifruit

**Figure 6:**
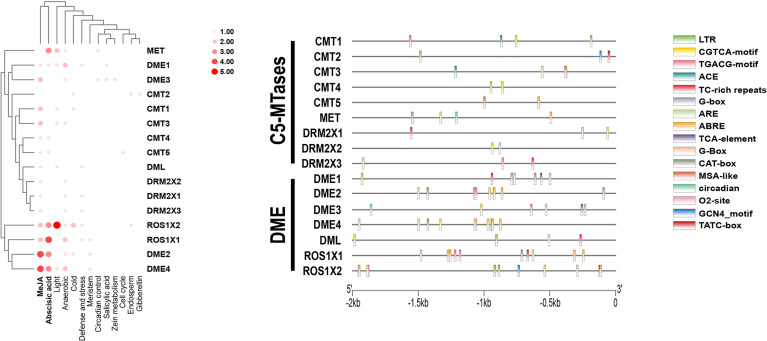
Cis-acting elements in the promoters of C5-MTases and demethylases.

The 2000 bp of kiwifruit genomic DNA sequence upstream of the transcriptional start sites of C5-MTases and DNA demethylases in kiwifruit were analyzed and *Cis*-acting elements of all promoters are shown in **Supplementary Data 5**. All promoter sequences contained a CAAT-box, a conventional cis-element. More specifically, most C5-MTases and DNA demethylases promoters contained the jasmonic acid-inducible CGTCA-motif, TGACG-motif, and abscisic acid-induced ABRE, and anaerobic inducing elements (ARE) (**Figure 6**). This suggests that C5-MTases and DNA demethylases may be responsive to both biotic and abiotic stressors, which is consistent with previous studies in peanuts (Wang et al., 2016).

### Expression of C5-MTases and DNA Demethylases in Kiwifruit

**Figure 7:**
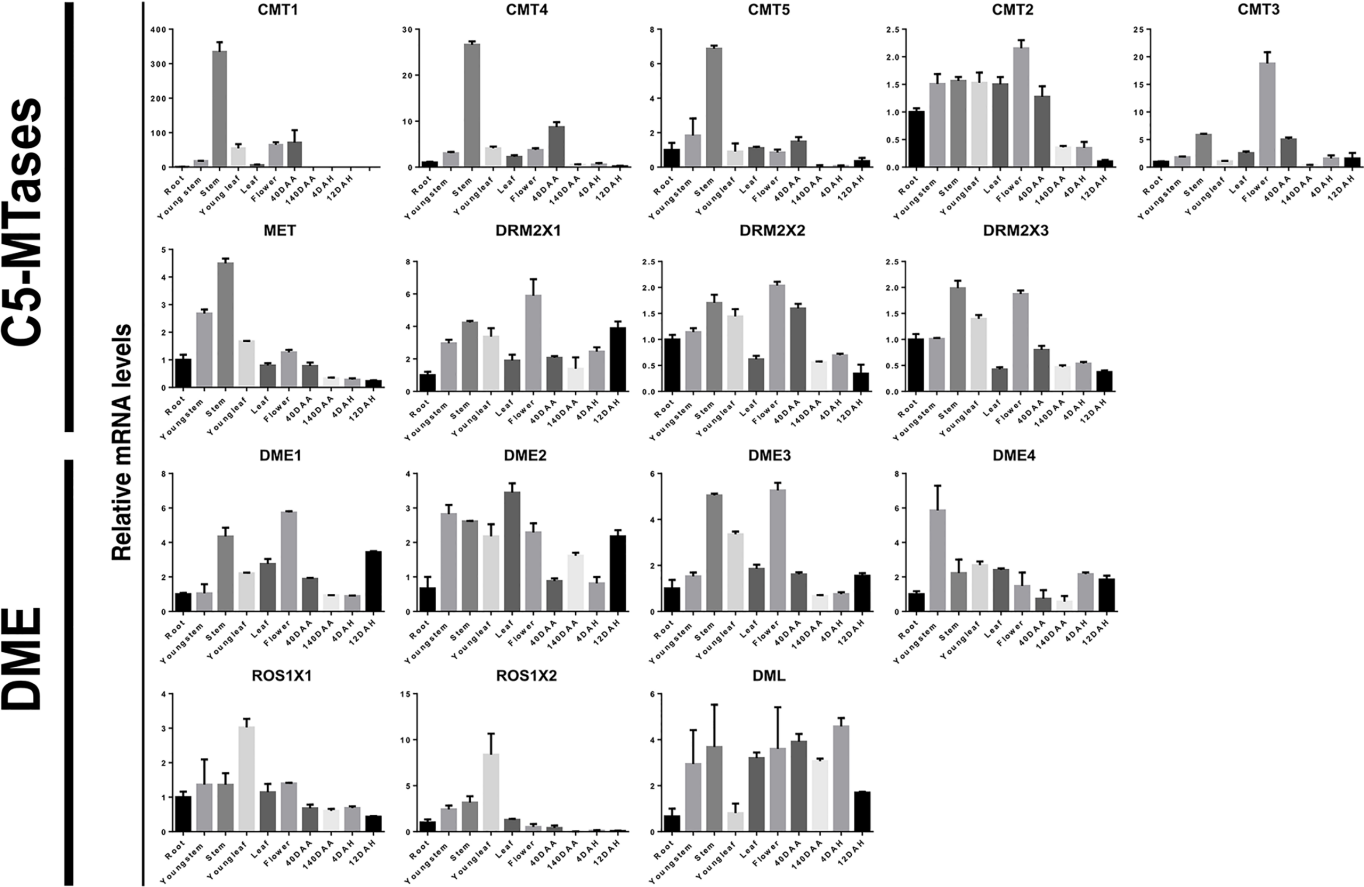
Relative expression levels of kiwifruit C5-MTase and demethylase genes in different tissues. 40DAA: Fruit 40 days after anthesis; 140DAA: Fruit 140 days after anthesis; 4DAH: Fruit 4 days after harvest; 12DAH: Fruit 12 days after harvest. Values represent the average of three biological replicates, and vertical lines represent the SD of the average.

C5-MTases are responsible for the establishment and maintenance of DNA methylation (Niederhuth and Schmitz, 2013). DNA demethylases, on the other hand, plays a major role in the process of DNA demethylation (Zhu, 2009). For further exploration of their putative role in plant growth and development, using qRT-PCR we analyzed the expression patterns of C5-MTases and DNA demethylases in different tissues and ripening stages including roots, young stems, stems, young leaves, leaves, flowers, fruit at 40 days after anthesis, fruits at 140 days after anthesis, fruits at 4 days after harvest, fruits at 12 days after harvest (**Figure 7**).

It is noteworthy that MET, CMT1, CMT4, and CMT5 have similar expression patterns. These genes are homologous and are all highly expressed in the stems of kiwifruit plants. However, their expression levels were relatively low during early developmental of the stem, suggesting that a large amount of DNA methylation only occurs for a certain period during plant stem development. The expression of CMT2 and CMT3 was more prominent in flowers and DRM2X1, DRM2X2, as well as DRM2X3 were more prominently expressed in stems and flowers than in early developmental stages. Interestingly, the expression of DRM2 were more prominent in fruit than that of MET and CMT. In particular, although DRM2X1, DRM2X2, and DRM2X3 are homologous genes, the expression pattern of DRM2X1 in fruits was different to that of DRM2X2 and DRM2X3. Relatively high levels of DNA demethylases were found in young stems, stems, young leaves, and flowers, but transcripts of DNA demethylases were rather low in roots.

## Discussion

In plants, DNA methylation has been figured out to be involved in different processes of plant growth and development (Hsieh and Fischer, 2005; FitzGerald et al., 2008; Yelina et al., 2015). Despite the fact that C5-MTases and DNA demethylases play a fundamental role in the determination of DNA methylation pattern in the epigenome, little is known about the control of DNA methylation status in kiwifruit. In our study, nine C5-MTases and seven DNA demethylases were identified in kiwifruit.

Phylogenetic analysis of C5-MTases from four plant species showed that the duplication of C5-MTases might be different between dicotyledonous and monocotyledonous plants (**Figure 1**). As shown in **Figure 2**, the C5-MTases of kiwifruit could be clearly divided into three groups: MET, CMT, and DRM2. Compared with other CMT members, CMT5 lacked part of the BAH domain which contained aromatic cages comprised of three conserved amino acids (Y, W, Y) capturing methylated-lysine.

**Figure 8:**
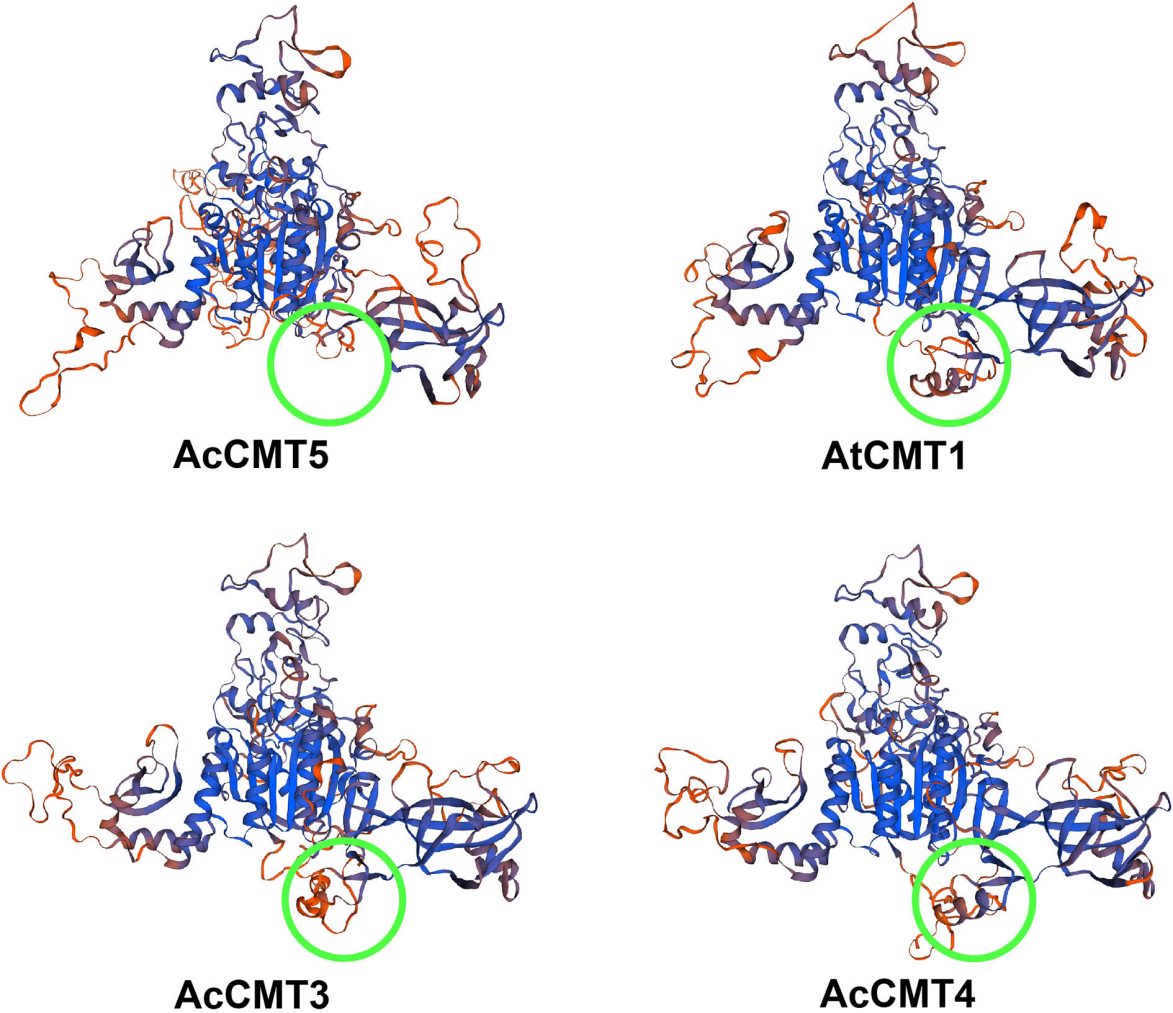
3D structure of AtMET1, AcMET3, AcMET4, and AcMET5.

To further investigate the difference in structures between CMT5 and other CMTs in kiwifruit, we built 3D models for AcCMT3, AcCMT4, AcCMT5, and AtCMT1 (**Figure 8**). From these models, we found that the BAH domain of AcCMT5 was incomplete and part of the domain was missing when compared with the BAH structural domain of AcCMT3 and AcCMT4. This suggests that the ability of AcCMT5 to bind methylated lysine might be different from the other CMTs.

Analysis of *cis*-acting elements has shown that C5-MTase and DNA demethylase genes are strongly associated with biological and abiotic stressors. This is consistent with changes in the expression of C5-MTase and DNA demethylase genes after kiwifruit is harvested, suggesting that postharvest storage might be affected by DNA methylation. Based on chromosomal localization and collinear analysis, we found that these gene pairs (DRM2X1, DRM2X2, DRM2X3; CMT4, CMT4, CMT5; CMT2, CMT3; DME2, DME3; DML and ROS1X2) in C5-MTases and DNA demethylases in kiwifruit are homologous. Their positions on chromosomes are similar, which is likely related to the three chromosome multiplication events of kiwifruit over the course of its evolutionary history. Through selection pressure analysis, we found these enzymatic proteins are strongly regulated under purifying selection and play an important conserved role in these organisms. Gene expression analysis of C5-MTases and DNA demethylases across different tissues and developmental stages showed that C5-MTases were more prominent in stems and flowers, while DNA demethylases had higher expression in young stems, stems, young leaves, and flowers. This suggests that DNA methylation dynamics are important in both vegetative and reproductive development.

## Conclusion

In our study, nine C5-MTases and seven DNA demethylases were identified in the kiwifruit genome. Through a comprehensive analysis of its gene structure, chromosome localization, and protein structure, we found similar results to C5-MTases and DNA demethylases previously recognized in *Arabidopsis*. Additionally, we found that expression level of DNA methylases were more prominent in stems and flowers, and DNA demethylases were expressed in the young stems, stems, young leaves, and flowers of kiwifruit. We also found that kiwifruit maintained a certain level of expression of C5-MTases and DNA demethylases after harvest, suggesting that DNA methylation dynamics may affect fruit ripening and postharvest. These results help to understand the complexity of these two gene families and lay a foundation for analyzing the role of DNA methylation modification in fruit ripening and fruit shelf life of kiwifruit.

## Data Availability Statement

All datasets generated for this study are included in the article/supplementary material.

## Author Contributions

ML and YZ designed the experiments. YZ, XH, and WX interpreted the results. YZ, XH, HZ, and HD wrote the paper. WX and SW participated in the data mining. XH, HW, and DS helped in kiwifruit materials collection and qRT-PCR analysis. ZZ, BY, JW, and DG helped improve the manuscript. All authors contributed to the article and approved the submitted version.

## Funding

This research was supported by the National Key R&D Program of China (2016YFD0400100), the Science and technology innovation talent project of Sichuan province (2018RZ0144) to ML and National Natural Science Foundation of China (No. 30670204) to JW. This project is also supported by the College student innovation and entrepreneurship training program of Sichuan University (201910610874).

## Conflict of Interest

Authors ZZ and BY were employed by company Sichuan Dexin Guoyuan Biological Technology Co., Ltd.

The remaining authors declare that the research was conducted in the absence of any commercial of financial relationships that could be construed as a potential conflict of interest.
